# Atomic structural details of a protein grafted onto gold nanoparticles

**DOI:** 10.1038/s41598-017-18109-z

**Published:** 2017-12-20

**Authors:** Stefano Giuntini, Linda Cerofolini, Enrico Ravera, Marco Fragai, Claudio Luchinat

**Affiliations:** 1 Department of Chemistry “Ugo Schiff”, University of Florence, Via della Lastruccia 3, 50019 Sesto Fiorentino, Italy; 2 Magnetic Resonance Center (CERM), University of Florence and Consorzio Interuniversitario Risonanze Magnetiche di Metallo Proteine (CIRMMP), Via L. Sacconi 6, 50019 Sesto Fiorentino, Italy; 3 GiottoBiotech S.R.L., Via Madonna del Piano 6, 50019 Sesto Fiorentino, Italy

## Abstract

The development of a methodology for the structural characterization at atomic detail of proteins conjugated to nanoparticles would be a breakthrough in nanotechnology. Solution and solid-state NMR spectroscopies are currently used to investigate molecules and peptides grafted onto nanoparticles, but the strategies used so far fall short in the application to proteins, which represent a thrilling development in theranostics. We here demonstrate the feasibility of highly-resolved multidimensional heteronuclear spectra of a large protein assembly conjugated to PEGylated gold nanoparticles. The spectra have been obtained by direct proton detection under fast MAS and allow for both a fast fingerprinting for the assessment of the preservation of the native fold and for resonance assignment. We thus demonstrate that the structural characterization and the application of the structure-based methodologies to proteins bound to gold nanoparticles is feasible and potentially extensible to other hybrid protein-nanomaterials.

## Introduction

Protein-inorganic conjugate nanomaterials find increasing applications in nanotechnology, cell biology and medicine^1–4^. The intrinsic hybrid nature of these materials is the major strength of their use, but also hampers the structural characterization at atomic detail of the different components when assembled together. Among these hybrid nanomaterials, specific proteins conjugated to gold nanoparticles are well-established tools for immunohistochemistry and promising agents for photodynamic therapy, near-infrared optical imaging and drug delivery^5–8^. Highly stable and biocompatible gold nanoparticles (GNPs) are obtained by grafting long polyethylene glycol (PEG) chains onto the surface of the inorganic core in order to decrease the uptake by reticuloendothelial system cells and increase the circulation half-life^9,10^. The use of bifunctional PEG chains bearing a gold-reactive group at one end and a reactive moiety at the other allows for the synthesis of PEGylated gold nanoparticles functionalized with biologically relevant proteins^11,12^. The covalent linkage of the protein is often achieved through the formation of an amide bond with the ε-amino groups of at least one surface exposed lysine residue^13^. This simple and versatile synthetic strategy leads to the conjugation of the protein at different sites and to the possibility of binding several protein molecules per particle. Solution NMR investigations on functionalized nanoparticles have been already reported for small molecules and peptides covalently linked through flexible spacers^14–16^. More recently, also the potentiality of solid-state NMR has been used to characterize nanoparticles bearing relatively small functionalities on the surface^17–19^. Conversely, the use of SSNMR to obtain atomic structural details on proteins grafted onto GNPs has never been described before. Therefore, the functional integrity of the proteins conjugated to GNPs is usually assessed by biochemical assays, assuming that the structure is preserved if the functionality is preserved. However, structural biology tools can provide a wealth of information that can be applied to the proteins grafted onto nanoparticles. Heteronuclear single quantum coherence (HSQC)^20^ correlating nitrogen and amide proton on ^15^N isotopically enriched proteins or acquired at natural abundance is a relatively simple and rapid experiment that is an extremely faithful reporter of minor changes occurring in the chemical environment of the observed nuclei. It is thus widely used (i) to assess the protein folding, (ii) to observe structural changes, and (iii) to monitor chemical modifications or protein-protein and protein-ligand interactions^21–24^, and it may be ported to larger systems through tailored sequences, even if the achievable size is still low^25,26^. More recently, amide proton-nitrogen correlation spectra have been reported on crystalline protein samples at the solid state^27–32^. However, high quality solid state 2D ^1^H-^15^N CP-HSQC can be recorded only at high magnetic fields, under fast magic angle spinning (MAS) conditions (60 kHz), possibly on perdeuterated samples to abolish the detrimental effects of proton-proton dipolar coupling on spectral resolution.

The molecular weight is not a limiting factor for solid-state NMR, and also non-crystalline samples can provide high quality spectra^33–37^.

We here show that the solid-state version of the HSQC experiment can be used to structurally characterize proteins covalently linked to GNPs. We have synthesized protein-GNPs starting from commercially available PEGylated GNPs with a core size of 5 nm, and the therapeutically relevant protein *E. coli* asparaginase II (ANSII). This protein has been already investigated in the solid state both in its native form, after PEGylation (PEG-ANSII hereafter)^38^ and after conjugation with polysaccharides^39^. The conjugation with the protein affects neither the stability of the GNPs in solution, nor their maximum absorption wavelength. The protein is only a fraction of the whole sample filling the 1.3 mm rotor, and therefore proton detection under fast MAS is necessary to achieve enough sensitivity. Highly resolved SSNMR spectra from ANSII conjugated to GNPs (ANSII-GNPs, hereafter) have been obtained after rehydration of freeze-dried material.

**Figure 1:**
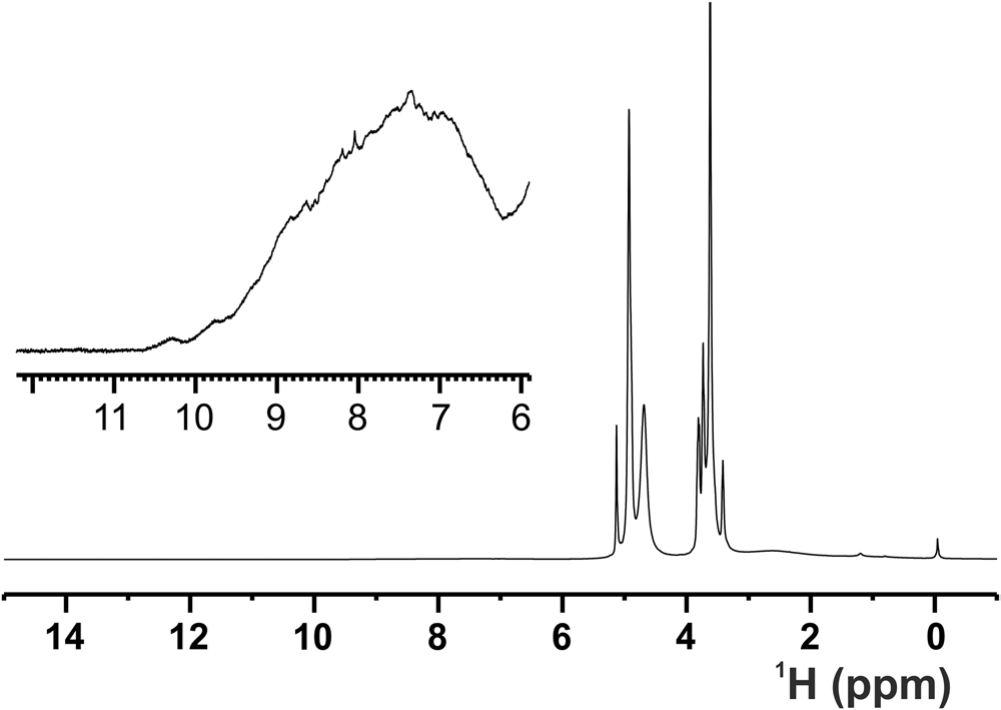
1D ^1^H NOESY NMR spectrum (mixing 20 ms) acquired at the solid-state on the ANSII-GNPs sample using a spectrometer operating at 800 MHz, ^1^H Larmor frequency, at ~282 K and MAS of 60 kHz. The sharp signals of the methylene group of the PEG chains and of the water are visible around 3.6 and 4.9 ppm, respectively. The top panel displays an enlargement, with a different intensity scale, of the region between 6 and 12 ppm, where the protein amide proton resonates. The high spreading of these signals is a positive marker of the protein folding.

**Figure 2:**
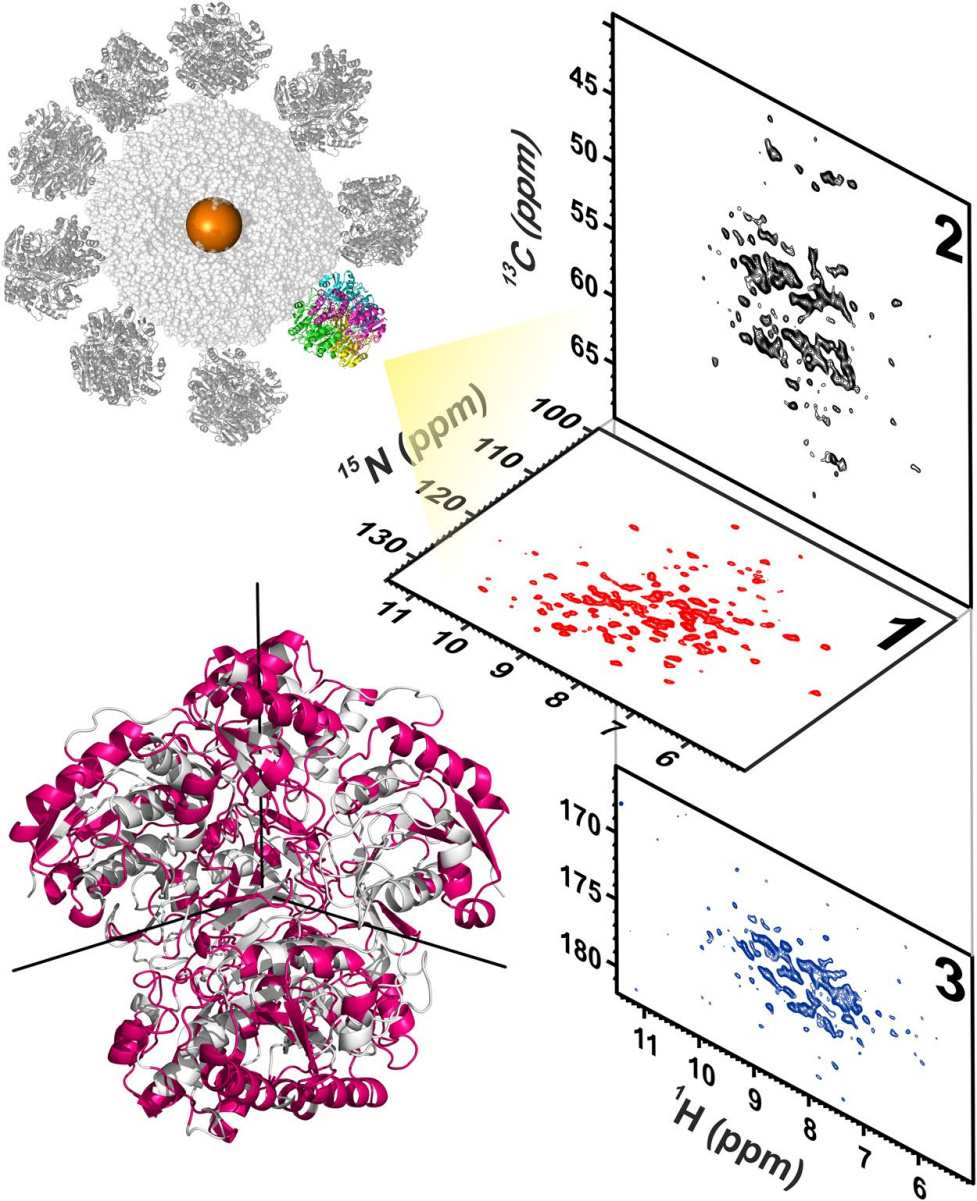
2D ^1^H-^15^N CP-HSQC SSNMR spectrum (1) and 2D ^1^H-^13^C planes of the 3D (H)CANH (2) and 3D(H)CONH (3) spectra obtained from ANSII-GNPs. The experiments were acquired on a spectrometer operating at 800 MHz, ^1^H Larmor frequency, at ~282 K and MAS of 60 kHz. Cartoon representation of tetrameric ANSII with highlighted in magenta the assigned residues in the 2D ^1^H-^15^N CP-HSQC SSNMR spectrum of ANSII-GNPs. The three C_2_ symmetry axes defining the D_2_ symmetry are displayed as black lines on the protein structure.

**Figure 3:**
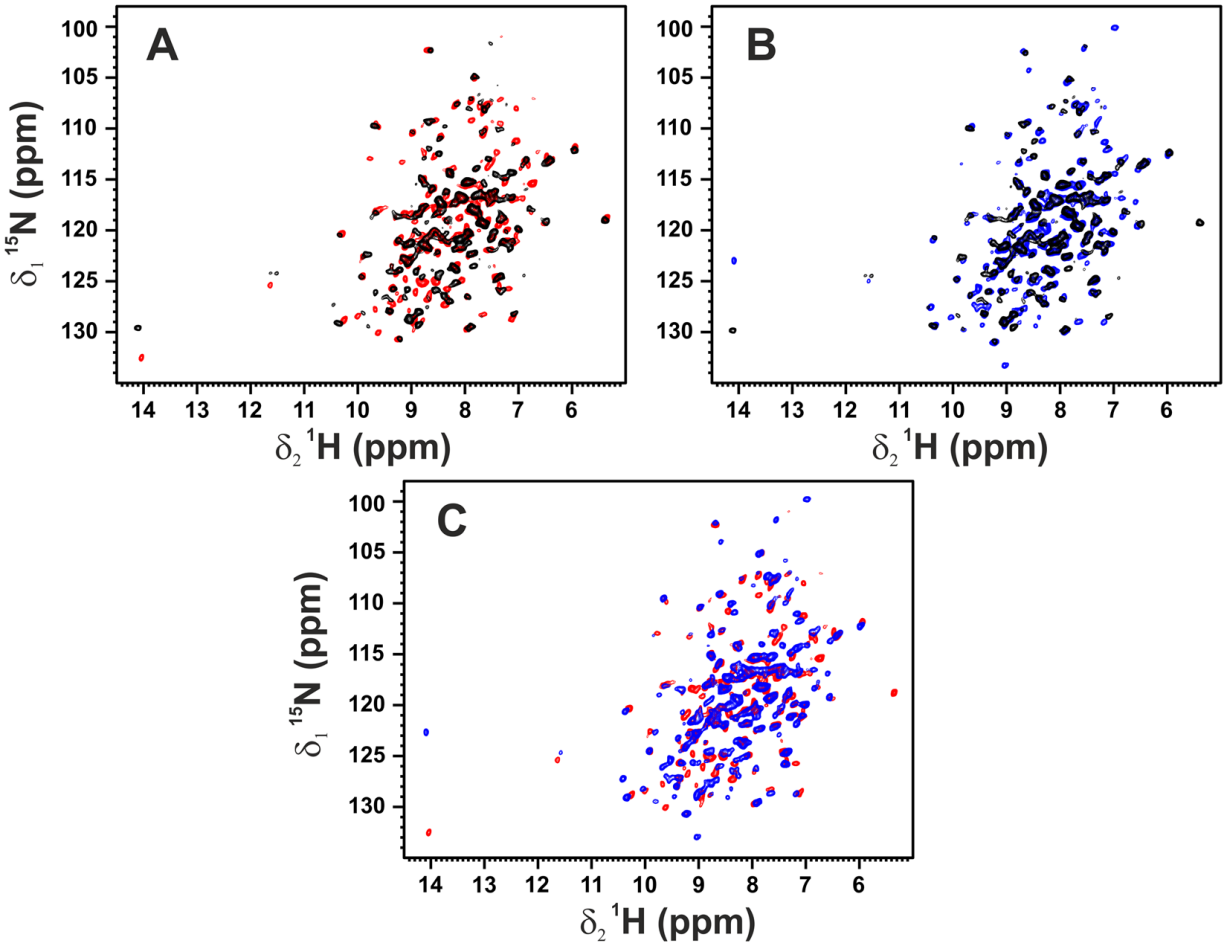
2D ^1^H-^15^N CP-HSQC SSNMR spectrum of ANSII-GNPs (black) superimposed with the spectrum of crystalline ANSII (red) (panel A), and PEG-ANSII (blue) (panel B). The superimposition of the spectra of crystalline and PEG-ANSII is also reported (panel C). The spectrum of ANSII-GNPs was acquired on a 800 MHz spectrometer, while the spectra of crystalline ANSII and PEG-ANSII were acquired on a 850 MHz spectrometer. All the spectra were recorded at ~282 K and MAS of 60 kHz.

The 1D ^1^H NOESY of ANSII-GNPs shows a large group of signals in the region between 6.8 and 10.5 ppm, and one very intense peak around 3.6 ppm (Fig. 1). The chemical shift values in the 6.8–10.5 ppm range are typical for protein amides protons, while the intense peak observed at 3.6 ppm nicely matches with the methylene signals of the PEG forming the coating of the investigated nanoparticles. The large chemical shift dispersions of the amide protons and the presence of signals downfield of 8.5 ppm are positive markers of the protein folding and prompt the application of heteronuclear correlation experiments. The 2D ^1^H-^15^N CP-HSQC SSNMR of ANSII-GNPs (Fig. 2, panel 1) is of very good quality, and comparable to the 2D ^1^H-^15^N CP-HSQC spectra collected on PEG-ANSII and on the crystalline preparation of the free ANSII (Fig. 3). In the three spectra the resonances are largely superimposable, immediately demonstrating that the native three-dimensional structure of the protein is preserved after the conjugation with the nanoparticles and PEG^38^. Furthermore the spectra are sufficiently resolved so that 184 amide signals over a total of 224 visible cross-peaks can easily be assigned by comparison with the spectra of both the crystalline and PEGylated preparations of ANSII for which assignment is available (unpublished data), and by the analysis of the ^1^H-^13^C planes of 3D (H)CANH and 3D(H)CONH spectra acquired on the ANSII-GNPs (Fig. 2, panels 2 and 3).

**Figure 4:**
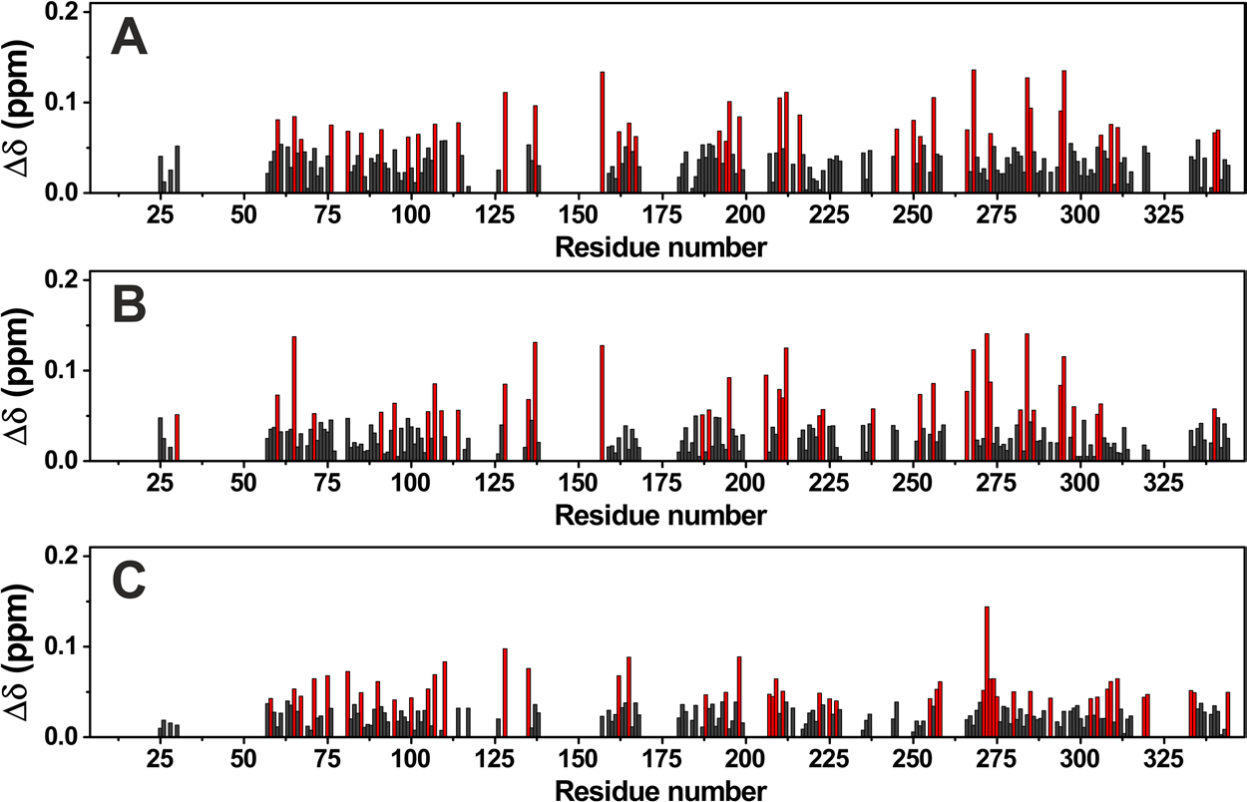
Chemical-shift perturbations of PEG-ANSII (**A**) and ANSII-GNPs (**B**) with respect to the crystalline preparation, and between ANSII-GNPs and PEG-ANSII (**C**), according to the formula ${\rm{\Delta }}\delta =\frac{1}{2}\sqrt{{\rm{\Delta }}{\delta }_{H}^{2}+{(\frac{{\rm{\Delta }}{\delta }_{N}}{5})}^{2}}$ ^40^. The residues experiencing the highest perturbation are highlighted in red.

The effects of conjugation with the PEGylated nanoparticles were investigated by observing the differences in chemical shift of the resonances in the spectra of ANSII collected at the solid state for the different protein preparations (crystalline free ANSII, PEG-ANSII and ANSII-GNPs). The chemical shift perturbation analysis of the 2D ^1^H-^15^N CP-HSQC spectrum of ANSII-GNPs (Fig. 4) reveals that the largest variations involve the residues located on the protein surface or on loops. This finding is not surprising because the effect of packing forces in the crystalline free ANSII, and the chemical modifications in PEG-ANSII involve residues at the protein surface. For most of the residues the chemical shift values of ANSII conjugated to GNPs are in better agreement with those of the PEG-ANSII than with the crystalline free ANSII, and this remarks the similarity between PEG-conjugated samples. Interestingly, the chemical shift variations are larger for the PEG-ANSII, consistently with a lower level of conjugation of the lysine residues on the protein grafted onto the PEGylated GNPs. In the PEG-ANSII sample 7 lysine residues are conjugated on average, whereas virtually only a single one of the 19 exposed lysine residues at a time is reacted to the PEG-coated nanoparticle.

In summary, we demonstrate that highly resolved heteronuclear spectra can be recorded on protein-GNPs and assigned to obtain structural information at atomic detail. This new evolution of the NMR methodology opens new frontiers for the development of the structure-based strategies applied to nanoparticles and potentially to all nanomaterials functionalized with proteins. The accessibility to all the experiments previously used only for pure protein samples represents a breaking point for the engineering and GNPs applications. The simplicity of the procedures for sample preparation, the exquisite sensitivity of magnetic resonance to structural perturbations, and the possibility to obtain a full structural characterization of the proteins conjugated on the GNPs, are important features of this methodology.

An additional benefit is the possibility of monitoring protein-protein interactions involving the biomolecule immobilized onto the GNPs surface. Moreover, this strategy can be extended to GNPs of different sizes and shapes since they retain the same chemical and structural features. However, we should point out that the signal to noise ratio is obviously a function of the protein-GNPs mass ratio, which is strongly correlated to the surface to volume ratio of the nanoparticles. Finally, this methodology is also directly applicable to nanoparticles with a different core composition if they are stable under MAS conditions, and if their use is not prevented by a fast MAS inside high magnetic fields.

## Methods

### Expression and purification of ANSII [U- ^2^H-^13^C-^15^N]

Escherichia coli C41(DE3) cells were transformed with pET-21a(+) plasmid encoding ANSII gene. The cells were cultured in ^2^H^13^,C^15^,N-enriched Silantes OD2 medium supplied with 0.1 mg mL^−1^ of ampicillin, grown at 310 K, until A600 nm reached 0.6–0.8, then induced with 1 mM isopropyl β-D-1-thiogalactopyranoside. They were further grown at 310 K overnight and then harvested by centrifugation at 7500 rpm (JA-10 Beckman Coulter) for 15 min at 277 K. The pellet was suspended in 10 mM Tris-HCl, pH 8.0, 15 mM EDTA, 20% sucrose buffer (60 mL per liter of culture) and incubated at 277 K for 20 min upon magnetic stirring. The suspension was centrifuged at 10000 rpm (F15–6 × 100 y Thermo Scientific) for 30 min and the supernatant discarded. The recovered pellet was re-suspended in H_2_O milli-Q (60 mL per liter of culture) and newly incubated at 277 K for 20 min under magnetic stirring. Again the suspension was centrifuged at 10000 rpm (F15–6 × 100 y Thermo Scientific) for 30 min. The pellet was discarded, whereas the supernatant treated with ammonium sulfate. Still under magnetic stirring solid ammonium sulfate was added in aliquots up to 50% saturation. The precipitate was removed by centrifugation, then further ammonium sulfate was added up to 90% saturation to trigger the precipitation of ANSII, which was recovered again by centrifugation. The precipitated ANSII was re-dissolved in a minimal amount of 20 mM Tris-HCl, pH 8.6 buffer and dialyzed extensively against the same buffer. ANSII was purified by anionic-exchange chromatography using a HiPrep Q FF 16/10 column (GE Healthcare Life Science). The protein was eluted in 20 mM Tris-HCl, pH 8.6 buffer with a linear 0–1 M NaCl gradient. Fractions containing pure ANSII were identified by Coomassie staining SDS-PAGE gels, then joined and dialyzed extensively against 50 mM phosphate, pH 7.5 buffer.

### Conjugation of ANSII [U- ^2^H-^13^C-^15^N] to 5 nm gold nanoparticles and sample preparation for SSNMR

A sample of ANSII [U- ^2^H-^13^C-^15^N] has been conjugated to gold nanoparticles with core size of 5 nm functionalized with 5 kDa PEG chains and bearing NHS-ester (~1 NHS group/nm^2^) to react with the protein lysine residues (Cytodiagnostic). ANSII is a tetrameric assembly of 138 kDa formed of four identical subunits organized as a dimer of dimers. The molecular weight of the ANSII is of the same order of the monoclonal antibody used for the optimization of the conjugation strategy with these nanoparticles. The experimental conditions used in the protein-GNPs synthesis allow us to optimize the protein loading, avoid protein-mediated GNPs aggregation, and minimize the amount of protein needed for the reaction. 200 μL of 20 mg mL^−1^ ANSII [U- ^2^H-^13^C-^15^N] solution in 50 mM sodium phosphate, pH 7.5 buffer were diluted up to 400 μL with the Protein Re-suspension Buffer, then further diluted up to 900 μL with the Reaction Buffer. The solution was splitted in ten 90 μL aliquots which were directly transferred in ten vials containing lyophilized NHS-activated gold nanoparticles. The reaction mixtures were immediately mixed by pipetting, incubated at room temperature for 2 hours, then quenched with 10 μL (per vial) of Quencher Solution. Protein-GNPs nanoparticles were purified from the unreacted protein by multistep washing on a centrifugal device with membrane nominal pore size of 10 nm (MWCO Microsep Advanced Centrifugal Device, Pall Corporation). Three washings were performed using 20 mM Tris-HCl, pH 8.0 buffer until all the free protein was reduced (see Figure S1), and at the end the volume was reduced down to 500 μL, splitted in 10 new 1.5 mL eppendorfs, frozen with liquid nitrogen and lyophilized. The freeze-dried sample, corresponding approximately to about 0.2 mg of protein, was packed on 1.3 mm rotor for a preliminary NMR analysis, but rehydration was needed to collect well resolved spectra.

### NMR measurements

All SSNMR spectra were recorded at ~282 K on Bruker AvanceIII HD spectrometer operating at 800 MHz ^1^H Larmor frequency, equipped with a 1.3 mm HCN probe-head, at MAS frequency of 60 kHz, using the standard parameters reported in literature^33,34^.

The nonselective 90° pulses were set to 2.05 μs (^1^H), 4 μs (^15^N), and 2.8 μs (^13^C).

^1^H-^15^N forward cross-polarization (CP) was achieved using a contact time of 1 ms (ω_H_ = 50 kHz, ω_N_ = 10 kHz) with a 70–100 linearly ramped contact pulse on ^1^H. The ^15^N-^1^H back CP was achieved using a contact time of 0.4 ms, (ω_H_ = 50 kHz, ω_N_ = 10 kHz) with a 100–70 linearly ramped contact pulse on ^1^H. The CP-HSQC was acquired with 2048 scans and with 156 increments in the indirect ^15^N dimension.

The ^1^H-^13^C forward CP had contact time of 3.5 ms for H-Cα and 2 ms for the H-CO transfer, respectively, with 70–100 linearly ramped contact pulses on ^1^H (ω_H_ = 40 kHz, ω_C_ = 20 kHz). CA-N CP and CO-N steps had contact time of 6 ms and 5 ms, respectively, with a 90–100 linearly ramped contact pulse of mean rf-field amplitude of about 40 kHz on ^13^C and a constant-amplitude spin lock of about 20 kHz on ^15^N.

All the spectra were with the Bruker TopSpin 3.2 software package and analysed with the program CARA (ETH Zürich).

## Electronic supplementary material

Supplementary Information
