# Supplementary material for: Atomic structural details of a protein grafted onto gold nanoparticles
Source: Sci Rep. 2017 Dec 20;7:17934. doi: 10.1038/s41598-017-18109-z (PMC5738368; doi:10.1038/s41598-017-18109-z)
Supplement: Supplementary file 1 — Supplementary Information [file 41598_2017_18109_MOESM1_ESM.doc]

Atomic structural details of a protein grafted onto gold nanoparticles

*Stefano Giuntini^§‡^, Linda Cerofolini^‡^, Enrico Ravera^§‡^*, Marco Fragai^§‡¥^*, Claudio Luchinat^§‡^**

§. Department of Chemistry, University of Florence, Via della Lastruccia 3, 50019 Sesto Fiorentino, Italy

‡. Magnetic Resonance Center (CERM), University of Florence and Consorzio Interuniversitario Risonanze Magnetiche di Metallo Proteine (CIRMMP), Via L. Sacconi 6, 50019 Sesto Fiorentino, Italy

¥ GiottoBiotech S.R.L., Via Madonna del Piano 6, 50019 Sesto Fiorentino, Italy

**SUPPLEMENTARY**

**INFORMATION**


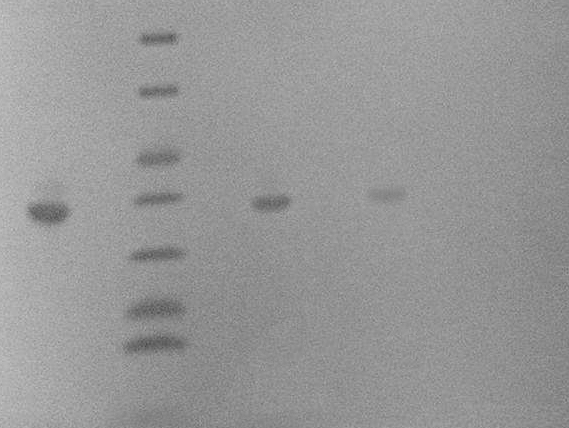


**1 2 3 4 5**

**35 kDa**

**25 kDa**

**18.4 kDa**

**14.4 kDa**

**45 kDa**

**66.2 kDa**

**116 kDa**

**Figure S1. Line 1** - pure ^2^H ^13^C ^15^N ANSII before conjugation reaction; **Line 2** - protein marker (kDa); **Line 3** - unbound protein after first washing; **Line 4** - unbound protein after second washing; **Line 5** – no unbound protein is detectable after the third washing

**Table S1.** ^1^H, ^15^N chemical shift of ANSII-GNPs. The spectra were collected at 800 MHz (^1^H Larmor frequency), at ~ 282 K and MAS of 60 kHz.

| **Res num** | **Res type** | **H** | **N** |
| --- | --- | --- | --- |
|  |  |  |  |
| 25 | ASN | 9.85 | 122.75 |
| 26 | ILE | 8.30 | 123.95 |
| 28 | ILE | 9.46 | 128.24 |
| 30 | ALA | 8.85 | 124.93 |
| 57 | LEU | 7.56 | 122.34 |
| 58 | VAL | 7.62 | 113.31 |
| 59 | ASN | 7.70 | 116.96 |
| 60 | ALA | 7.50 | 120.27 |
| 61 | VAL | 7.51 | 117.37 |
| 63 | GLN | 9.30 | 118.98 |
| 64 | LEU | 8.43 | 117.85 |
| 65 | LYS | 7.48 | 114.83 |
| 66 | ASP | 7.28 | 115.14 |
| 67 | ILE | 7.57 | 113.20 |
| 68 | ALA | 8.10 | 119.24 |
| 69 | ASN | 8.85 | 118.19 |
| 70 | VAL | 8.61 | 124.69 |
| 71 | LYS | 8.46 | 126.78 |
| 72 | GLY | 8.75 | 109.64 |
| 73 | GLU | 8.98 | 120.39 |
| 74 | GLN | 9.16 | 126.98 |
| 75 | VAL | 8.87 | 129.42 |
| 76 | VAL | 7.75 | 110.70 |
| 77 | ASN | 8.69 | 120.82 |
| 81 | GLN | 9.95 | 128.46 |
| 82 | ASP | 7.74 | 119.65 |
| 83 | MET | 7.28 | 120.46 |
| 84 | ASN | 6.44 | 113.47 |
| 85 | ASP | 8.81 | 117.92 |
| 86 | ASN | 8.02 | 115.55 |
| 87 | VAL | 7.56 | 122.21 |
| 88 | TRP | 7.35 | 121.42 |
| 89 | LEU | 8.19 | 117.47 |
| 90 | THR | 7.94 | 115.48 |
| 91 | LEU | 7.02 | 120.46 |
| 92 | ALA | 7.46 | 125.11 |
| 93 | LYS | 7.84 | 114.28 |
| 94 | LYS | 8.00 | 121.68 |
| 95 | ILE | 8.59 | 126.18 |
| 96 | ASN | 8.01 | 115.92 |
| 97 | THR | 8.37 | 116.64 |
| 98 | ASP | 8.26 | 121.24 |
| 99 | CYS | 7.49 | 120.49 |
| 100 | ASP | 8.53 | 112.81 |
| 101 | LYS | 8.05 | 117.15 |
| 102 | THR | 7.31 | 115.28 |
| 103 | ASP | 8.60 | 118.80 |
| 104 | GLY | 7.68 | 108.51 |
| 105 | PHE | 7.38 | 117.34 |
| 106 | VAL | 8.37 | 121.47 |
| 107 | ILE | 9.12 | 126.34 |
| 109 | HIS | 9.09 | 129.06 |
| 110 | GLY | 8.53 | 111.36 |
| 114 | MET | 7.33 | 126.19 |
| 116 | GLU | 7.89 | 122.73 |
| 117 | THR | 7.70 | 115.64 |
| 126 | LYS | 9.35 | 124.43 |
| 127 | CYS | 6.85 | 118.19 |
| 128 | ASP | 8.91 | 128.35 |
| 134 | VAL | 8.95 | 122.02 |
| 135 | GLY | 8.71 | 112.28 |
| 136 | ALA | 8.94 | 122.29 |
| 137 | MET | 11.58 | 124.53 |
| 138 | ARG | 9.98 | 124.92 |
| 157 | THR | 7.98 | 108.35 |
| 159 | ALA | 9.13 | 123.02 |
| 160 | ASP | 7.20 | 122.08 |
| 161 | LYS | 8.93 | 129.48 |
| 162 | ALA | 9.26 | 121.34 |
| 164 | ALA | 7.16 | 121.36 |
| 165 | ASN | 8.67 | 112.31 |
| 166 | ARG | 7.95 | 115.83 |
| 167 | GLY | 7.78 | 107.82 |
| 168 | VAL | 7.65 | 119.82 |
| 180 | ARG | 7.93 | 120.74 |
| 181 | ASP | 8.56 | 120.49 |
| 182 | VAL | 7.20 | 117.18 |
| 183 | THR | 8.79 | 121.84 |
| 184 | LYS | 7.73 | 128.78 |
| 185 | THR | 7.89 | 118.18 |
| 187 | THR | 7.56 | 117.14 |
| 188 | THR | 7.60 | 108.06 |
| 189 | ASP | 8.30 | 125.63 |
| 190 | VAL | 9.19 | 123.15 |
| 191 | ALA | 9.45 | 127.15 |
| 192 | THR | 7.15 | 111.83 |
| 193 | PHE | 9.45 | 124.40 |
| 194 | LYS | 8.55 | 122.49 |
| 195 | SER | 9.14 | 126.07 |
| 196 | VAL | 6.01 | 112.48 |
| 197 | ASN | 9.74 | 118.55 |
| 198 | TYR | 9.10 | 119.11 |
| 199 | GLY | 7.42 | 106.12 |
| 206 | ASN | 9.51 | 128.16 |
| 207 | GLY | 8.69 | 102.65 |
| 208 | LYS | 7.64 | 119.55 |
| 209 | ILE | 8.97 | 124.35 |
| 210 | ASP | 8.41 | 126.90 |
| 211 | TYR | 8.50 | 124.97 |
| 212 | GLN | 8.38 | 120.62 |
| 214 | THR | 8.32 | 114.96 |
| 217 | ARG | 8.04 | 115.83 |
| 218 | LYS | 7.35 | 121.46 |
| 219 | HIS | 7.88 | 115.52 |
| 220 | THR | 9.74 | 110.01 |
| 221 | SER | 8.48 | 118.56 |
| 222 | ASP | 8.93 | 120.65 |
| 223 | THR | 7.43 | 110.50 |
| 225 | PHE | 6.53 | 119.53 |
| 226 | ASP | 7.92 | 122.61 |
| 227 | VAL | 8.92 | 118.68 |
| 228 | SER | 8.27 | 117.72 |
| 235 | LYS | 9.13 | 123.60 |
| 236 | VAL | 9.40 | 125.99 |
| 237 | GLY | 8.68 | 116.35 |
| 238 | ILE | 9.73 | 118.59 |
| 244 | ASN | 7.93 | 117.90 |
| 245 | ALA | 5.43 | 119.38 |
| 250 | ALA | 7.51 | 117.17 |
| 251 | LYS | 8.55 | 117.51 |
| 252 | ALA | 8.52 | 118.63 |
| 253 | LEU | 10.39 | 120.78 |
| 255 | ASP | 9.00 | 122.94 |
| 256 | ALA | 7.36 | 118.66 |
| 257 | GLY | 8.17 | 107.97 |
| 258 | TYR | 7.99 | 120.57 |
| 259 | ASP | 8.07 | 121.02 |
| 266 | VAL | 8.42 | 110.13 |
| 268 | ASN | 9.85 | 129.79 |
| 269 | GLY | 7.87 | 105.18 |
| 270 | ASN | 9.02 | 123.18 |
| 271 | LEU | 8.52 | 118.06 |
| 272 | TYR | 7.89 | 123.83 |
| 273 | LYS | 7.12 | 128.58 |
| 274 | SER | 6.47 | 113.21 |
| 275 | VAL | 7.41 | 126.32 |
| 276 | PHE | 8.76 | 121.61 |
| 277 | ASP | 8.51 | 118.37 |
| 278 | THR | 7.53 | 116.73 |
| 279 | LEU | 8.68 | 122.06 |
| 280 | ALA | 9.40 | 123.86 |
| 281 | THR | 7.69 | 115.78 |
| 282 | ALA | 8.21 | 125.43 |
| 283 | ALA | 8.74 | 122.24 |
| 284 | LYS | 7.60 | 117.79 |
| 285 | THR | 7.75 | 107.82 |
| 286 | GLY | 7.55 | 107.57 |
| 287 | THR | 8.06 | 122.06 |
| 288 | ALA | 7.93 | 129.82 |
| 289 | VAL | 9.24 | 124.29 |
| 291 | ARG | 9.40 | 126.24 |
| 293 | SER | 8.84 | 115.31 |
| 294 | ARG | 9.72 | 126.42 |
| 295 | VAL | 8.94 | 124.61 |
| 297 | THR | 7.27 | 107.99 |
| 298 | GLY | 8.71 | 109.55 |
| 299 | ALA | 7.77 | 118.74 |
| 300 | THR | 7.93 | 121.67 |
| 301 | THR | 8.96 | 119.05 |
| 302 | GLN | 8.34 | 119.29 |
| 303 | ASP | 9.14 | 118.91 |
| 304 | ALA | 8.26 | 124.25 |
| 305 | GLU | 8.35 | 119.48 |
| 306 | VAL | 7.60 | 117.29 |
| 307 | ASP | 8.33 | 127.32 |
| 308 | ASP | 7.99 | 126.88 |
| 309 | ALA | 8.47 | 120.97 |
| 310 | LYS | 7.37 | 118.93 |
| 311 | TYR | 6.90 | 113.46 |
| 312 | GLY | 7.91 | 107.41 |
| 313 | PHE | 7.49 | 116.58 |
| 314 | VAL | 8.01 | 120.27 |
| 315 | ALA | 9.26 | 131.03 |
| 317 | GLY | 8.65 | 116.18 |
| 319 | LEU | 7.04 | 120.22 |
| 320 | ASN | 7.38 | 124.84 |
| 333 | THR | 7.71 | 110.55 |
| 334 | GLN | 8.11 | 119.26 |
| 335 | THR | 8.65 | 116.00 |
| 336 | LYS | 8.14 | 121.23 |
| 337 | ASP | 8.55 | 124.98 |
| 339 | GLN | 8.17 | 117.04 |
| 340 | GLN | 7.70 | 119.90 |
| 341 | ILE | 8.88 | 121.76 |
| 342 | GLN | 8.78 | 121.26 |
| 343 | GLN | 7.80 | 118.58 |
| 344 | ILE | 8.07 | 121.97 |
|  |  |  |  |
|  |  |  |  |
